# Seven-Site Facial Contouring with Botulinum Toxin Alone: A Practical Framework

**DOI:** 10.3390/toxins18100448

**Published:** 2026-10-15

**Authors:** Woon-Kyong Chung, Min Joo Kim

**Affiliations:** 1 You&Chung Skin Clinic, Seoul 06062, Republic of Korea; 2 V&MJ Dermatology Clinic, Seoul 06021, Republic of Korea; md.minjoo.kim@gmail.com

**Keywords:** botulinum toxin, facial contouring, masseter, temporalis, parotid gland, submandibular gland, platysma, depressor anguli oris, mentalis, jawline

## Abstract

More aesthetic patients now ask for changes in facial contour: a smaller face, a sharper jawline, and less sagging. Botulinum toxin (BoNT) is best known as a treatment for wrinkles, but it can also be used for modifying facial contour. With BoNT alone, a wide face can be made narrower, a heavy lower face can be made lighter, sagging on the jawline can be reduced, and a short chin can be corrected. Many physicians already do this, but each site is taught and studied on its own. This review brings the sites together under the name “contouring BoNT”. It describes seven sites: five muscles (masseter, temporalis, platysma, depressor anguli oris, mentalis) and two salivary glands (parotid, submandibular). The seven sites work in three ways: reducing the volume of a muscle or gland, removing a downward pull so that the face looks lifted, and relaxing an overactive muscle that changes the shape of the chin. All three serve one goal that patients describe in their own words: a smaller and lifted face. For each site, the review explains what it does to the contour, who is a good candidate, who needs caution, the published dose range and the authors’ practice, and which neighboring site changes when the target is treated. It explains which sites are checked together and how each is examined. Two safety points specific to contouring—the immunogenicity of large repeated doses, and salivary gland treatment—are discussed separately. The framework provided is a checklist for assessment, not a rule stating that every patient needs all seven sites to be treated.

## 1. Introduction

More and more aesthetic patients are requesting changes in facial contour, wanting a smaller face, a sharper jawline, less sagging of the lower face, or the correction of a short chin. This includes the large group who ask for “lifting” or “tightening”. Facial contour has become one of the main reasons for visiting an aesthetic clinic. In BoNT, this change is reflected in regulatory activity in the United States: platysma bands, a neck contour indication, were approved by the FDA in 2024 [1], and masseter muscle prominence is under FDA review after two phase 3 trials [2]. These approvals are specific to the United States; neither indication is approved in most other jurisdictions.

Contour deserves this attention. It is a large part of what makes a face look young or old. Studies of perceived age consistently rank sagging and facial shape together with wrinkles as signs of age, across ethnic groups [3,4,5]. In a recent study of East Asian women, the strongest signs of age were the nasolabial fold, the marionette fold, and a loss of contour tightness [6]. These are features of contour, not skin. A treatment that improves contour improves perceived age, even if it does not touch a wrinkle.

Patients know masseter BoNT well. Few know that BoNT can change other parts of the contour, and many physicians still think of it mainly as a wrinkle treatment. For the physician, it is a strong tool for this purpose, for a reason that does not depend on opinion: it is the only injectable that makes a muscle or a gland smaller, and the only one that removes a downward pull. Filler, threads, and energy devices add volume, lift tissue, or tighten skin; BoNT works on a different part of the problem, and the treatments complement each other. For patients whose contour problem is mainly a large muscle or gland, BoNT alone produces most of the desired result, quickly, at low cost, with no downtime, and reversibly. This is not a small group. In a survey of 469 Korean dermatologists, masseter treatment was among the most requested BoNT indications, and it was most popular in patients in their twenties and thirties [7]. The other sites in this review are treated for the same reason every day.

The literature on these sites is rich but scattered. There is a systematic review on the masseter, for which a phase 3 program is also underway. The temporalis, parotid gland, submandibular gland, depressor anguli oris, platysma and mentalis each have their own smaller literature, usually reviewed alone [8,9,10,11,12], and a recent review of contouring with neuromodulators covered the masseter, temporalis, and parotid [13]. What is missing is the step before the injection point: which site to treat in this patient, and why. This review provides that step. In this review, contouring BoNT means BoNT used to change the shape of the face at rest, rather than to remove a line. The review organizes the seven sites by how they work (Section 3), describes each site (Section 4), provides a short assessment guide and table (Section 5), and discusses the two safety points specific to contouring: immunogenicity and salivary gland treatment (Section 6). Published evidence and the authors’ own practice are kept separate throughout: the evidence for each site is summarized in Table 1, and the selection logic is presented as a proposed, expert-based framework that has not yet been tested prospectively.

## 2. Methods

This is a narrative review. We searched PubMed and Scopus from inception to September 2026 for clinical, anatomical, imaging, and safety studies of botulinum toxin injection into the masseter, temporalis, parotid gland, submandibular gland, depressor anguli oris, platysma, and mentalis for facial contour. The search terms combined “botulinum toxin” or “botulinum neurotoxin” with each site name and with “facial contouring”, “facial slimming”, “jawline”, “chin”, and “temple”. Reference lists of retrieved articles, consensus statements, and regulatory documents were searched by hand. We included randomized trials, prospective and retrospective studies, case series, case reports, anatomical and imaging studies, and consensus statements in English that addressed one of the seven sites as a contouring target or its safety. Studies of these sites for other indications (wrinkles, bruxism, pain, sialorrhea) were used only where they gave dose, safety, or volume data relevant to contouring. No formal quality scoring was applied; the type of evidence available for each site is summarized in Table 1.

The selection logic of the framework—who is a good candidate, who needs caution, and which sites are checked together—comes from the authors’ clinical practice in an East Asian population. It is labeled as the authors’ practice or observation wherever it appears, and it is proposed for prospective evaluation, not presented as established evidence.

## 3. How the Seven Sites Work

The seven sites were chosen based on three criteria: the structure shapes the contour of the face at rest; BoNT changes it in a way that is seen and reproducible in practice; and there is at least some published support for its use as a contouring target. Other structures were considered and left out. The buccinator and the lip elevators were excluded because weakening them harms oral function or facial expression for little contour gain. The risorius was excluded because its contour effect is small and the evidence is limited to anecdotes. Other facial muscles shape expression or wrinkles rather than the resting contour and were not considered. The list is therefore a practical checklist, not a complete map of the face, and it may grow as evidence grows.

The seven sites work in three ways:

**Reduce.** BoNT makes the injected structure smaller. There are four sites: two muscles, the masseter and the temporalis, and two glands, the parotid and the submandibular. The masseter and parotid make the lower face wide; the temporalis makes the upper face wide; the submandibular gland blurs the line between the jaw and neck.

**Rebalance.** BoNT turns off a muscle that pulls down, so that the muscles that pull up win. Two sites work as one system: the platysma and the depressor anguli oris. The facial part of the platysma runs deep to the depressor anguli oris into the lower lip and modiolus; treating the platysma along the jawline and the depressor anguli oris near the mouth corner removes the same pull at two points.

**Release.** BoNT relaxes an overactive muscle that deforms a shape. There is one site, the mentalis, which shortens and bunches the chin.

“Seven sites” does not mean seven injections. It means seven places to look. The physician looks at all seven and treats the ones that need treatment (Figure 1).

## 4. The Seven Sites

Table 1 summarizes the evidence available for each site. It differs widely: randomized trials and a phase 3 program have investigated the masseter; one randomized trial and prospective series have been conducted on the parotid; for the platysma, randomized trials have been conducted for bands and one prospective study for the jawline; the temporalis, submandibular gland, depressor anguli oris, and mentalis rest on small series, anatomical studies, and consensus. In the paragraphs that follow, “published doses” are the doses reported in the cited studies, with the product named there. “The authors use” refers to the authors’ starting doses in East Asian patients; these represent expert practice, not trial evidence, and they are not expected to transfer unchanged to other populations. Of the neighboring-site effects described under “What to watch”, the temporalis response to masseter treatment is documented in trials; the others are the authors’ observations.

### 4.1. Masseter

**What it does.** The masseter often forms the lateral boundary of the lower face in front of the mandibular angle. When it is large, the lower face looks square and heavy from the front. Reducing it narrows the lower face; a prospective pilot study using 3D surface measurement in 11 patients found the masseter area to be smaller at 1 month and still reduced at 3 months [17]. This is the most studied contouring site, with comprehensive reviews of technique and safety [18,19] and a phase 3 program under regulatory review [2].

**Who is a good candidate.** A patient whose lower-face boundary is muscle. This must be confirmed, not assumed. Ask the patient to clench; the masseter hardens, and the boundary becomes more prominent. Feel it with both hands. Younger patients with firm skin respond best because the skin follows the shrinking muscle.

**Who needs caution.** Three groups.

The first group has a wide lower face that is not mainly masseter. From the front, the lateral boundary may be formed by cheek soft tissue in front of the masseter (Figure 2) or by the parotid gland behind it. Masseter BoNT does little for either; the parotid needs its own treatment (Section 4.3), and cheek soft tissue is not a BoNT target.

The second group has loose skin. If the masseter shrinks quickly and the skin cannot follow, the result is jowl sagging, or a visible step at the anterior border of the masseter where the treated muscle meets untreated cheek.

The third group has a pre-existing lateral cheek hollow. If the cheek above the masseter already lacks volume, reducing the masseter also removes the volume below it, and the patient says that the cheek “sank” after treatment. This happens more when injections are placed high. Inject low, toward the jawline.

**How.** The published doses are mostly 20–30 U per side. The authors use 10–20 U per side, 3–4 points.

**What to watch.** Two things.

The temporalis. A randomized triple-blind trial found that patients who received a second masseter injection at 3 months had a thicker temporalis on ultrasound and higher temporalis EMG activity at 6 months than patients who received saline [14]. A later study found that the temporalis thickness rose from 9.3 to 13.1 mm at rest after masseter BoNT [20]. The practical rule: check the temporalis in every masseter candidate at baseline, and again at follow-up in patients treated repeatedly. If the temporalis is already large and bulges on clenching, treating both can be considered; otherwise, explain what may happen and reassess the temporalis at follow-up. The decision is made for each patient, not as a rule.

The patient who no longer needs it. After repeated treatment, the masseter often does not return to its original size, even long after the last injection [15,16]. Once this happens, treatment does not need to be repeated on schedule. In the authors’ experience, some patients nevertheless ask for it again and again while the muscle is no longer large. They should be examined and told when there is nothing left to reduce.

### 4.2. Temporalis

**What it does.** The temporalis forms the lateral boundary of the upper face. When it is large, the forehead looks wide, and the face takes an inverted-triangle shape. Reducing it narrows the upper face (Figure 3C,D). The circumference of the upper face decreases measurably after temporalis BoNT [21].

**Who is a good candidate.** Three groups. The first group is patients with an inverted-triangle face and a visible temporal bulge on clenching. The second group is every masseter candidate, as a baseline check (Section 4.1). The third group is patients asking for a smaller face in general. A useful clue: many women with a large temporalis wear their hair down over the temples. They usually know about the problem before the physician does.

Repeated masseter-only treatment in a patient with a large temporalis makes the inverted triangle worse over time. This is the clearest reason to assess the two muscles together.

**Who needs caution.** The patient with a temple hollow—not because BoNT is wrong for them, but because the two treatments of the temple go to different places. The hollow lies in the lower front part of the temporal fossa, where the surface layers are mainly fascia and fat over a thin, tendinous part of the muscle [42]; this is where filler goes. The muscle belly that BoNT treats lies higher and further back. Injecting low, in the hollow zone, does not narrow the face and can make the hollow look deeper, so the authors avoid it unless that area bulges on chewing.

**How.** The published doses vary widely and are mostly higher than those below [21,22]. The authors use 10–20 U per side, 4–5 points, into the muscle belly.

**What to watch.** Hollowing, when injections are placed too low, or the belly is over-reduced. Headache and chewing fatigue are uncommon.

**An observation not previously reported.** In patients who want temple filler for a hollow temple, the authors first check the temporalis muscle above the hollow. The hollow is partly a step between the hollow zone and the muscle belly above and behind it. When the belly is large, the step is deep and the hollow looks worse than its true volume loss. Reducing the belly with BoNT softens the step, and the amount of filler needed is then smaller—which matters, because filler placed in contour areas enlarges the face, and temple filler can require up to 2 mL per side [43]. It is established that temporalis size sets the outline of the upper face. The use of temporalis BoNT to reduce the amount of temple filler is the authors’ clinical observation; it has not been tested in a study.

### 4.3. Parotid Gland

**What it does.** The parotid sits behind the masseter, in front of and below the ear. When it is large, it forms part of the lateral boundary of the lower face, and it is often the real cause of a face that looks square but does not respond to masseter BoNT. BoNT reduces it: a randomized trial with high-dose, low-dose, and placebo arms found significant volume reduction in both treated arms, with recovery of only 5–8% at 3 months [23], and prospective series confirm the effect [10,24].

**Who is a good candidate.** A patient whose parotid is enlarged, whether or not the masseter is also large. When both are large, both are treated (Figure 3A,B). When only the masseter is treated in such a patient, the parotid becomes the new boundary, and the face looks no narrower. The gland does not harden on clenching and is soft on palpation with both hands. Population MRI data explain who these patients are: the mean parotid volume was 27.8 cm^3^ in men and 21.6 cm^3^ in women, and the volume rose with body mass index, age, and male sex [44]. In the authors’ experience, the pattern is clear: the gland is largest in overweight, older men.

There is one note on expectations. In overweight patients, part of the gland enlargement is fat, not secretory tissue. The parotid fat fraction rose from 30% in underweight to 47% in overweight subjects on MRI [45]. BoNT causes the atrophy of secretory tissue and does not remove fat. A larger gland needs a larger dose, and the secretory part will shrink in proportion. But the fat will not, so the gland does not shrink as much as its size suggests. The patient should be told this before treatment.

**Who needs caution.** A patient with a gland that is not clinically evident.

**How.** Published protocols vary in dose and point number [10,23,24]. The authors use 15–40 U per side, higher in very large glands. The number of points follows the shape of the enlargement: 2–3 points when only the part in front of the ear is prominent, and 5–8 points when both the parts in front of and below the ear are enlarged. Ultrasound guidance is advisable, particularly for less experienced injectors (Section 6.2). A practical point: the gland stands out more when the patient lies back, so the authors inject reclined, where the outline of the gland is easier to follow.

When the masseter and parotid are both treated, each is dosed based on its own criteria.

**What to watch.** Dry mouth after parotid BoNT is uncommon in the authors’ experience, and the published cosmetic parotid series and trial reported no serious adverse events [23,24]. The physiological reason is that, at rest, most saliva comes from the submandibular glands, while the parotid contributes mainly stimulated flow during eating [46]. Direct measurements of dry mouth after cosmetic parotid treatment are lacking.

The doses used for contouring lie within the range approved for sialorrhea (Section 6.2).

### 4.4. Submandibular Gland

**What it does.** The submandibular gland lies in the submandibular triangle, below the mandibular border and in front of the mandibular angle, under the platysma [25,27]. When the gland is prominent, it blurs the line between the jaw and neck and can look like excess fat under the jaw. Reducing it sharpens that line.

**Who is a good candidate.** Two different patients, same site.

The young patient with a small mandible. When the jaw is short and the jawline is underdeveloped, a normal-sized gland sits exposed below the mandibular border. CT data confirm that many young subjects have a gland 20 mm or more below the mandibular border [28]. Reducing the gland helps. In these patients, jawline filler is the more active option, and the two can be combined.

The older patient with a ptotic gland. The gland does not grow with age; it descends. Its distance below the mandibular border increases with age, about 0.17 mm per year, while its total volume does not change [28]. Imaging confirms that the part of the gland below the mandible grows with age while the whole gland does not [29]. Age-related submandibular fullness is therefore ptosis, not enlargement. BoNT reduces the bulk of the exposed part. It does not lift the gland, and the patient should know this.

Many patients take the submandibular gland that blurs their jawline for fat, and ask for fat reduction. On examination, the fullness is often the gland, or the gland with fat over it. The gland is felt as a firm, separate lobule below the mandibular border. When both are present, both are treated.

**A practical point for the injection, not described to the authors’ knowledge.** The gland is seen in the consultation with the patient upright, and what is seen there is what is treated. On the treatment bed, it can become less prominent, or disappear, once the patient lies down. Injecting with the patient as upright as possible keeps the gland where it was seen.

**Who needs caution.** A patient with a gland that cannot be felt. Contouring treats what shows.

**How.** A published protocol uses 20 U in 0.2 mL at five points 5 mm apart under ultrasound, with the gland diameter falling from 15 to 10 mm at 2 months [25]; a single-point ultrasound-guided protocol uses 15 U [26]. The authors use 10–15 U per side, 1–2 points, a high concentration, and a small volume, and do not go above this range. Ultrasound guidance is the standard precautionary approach for this gland (Section 6.2).

**What to watch.** Dry mouth and difficulty swallowing. This is the gland that produces most resting saliva, so dry mouth is more likely than after parotid treatment; in the authors’ experience, it occurs in approximately one in ten patients and is mild and temporary. Dysphagia has followed the spread of the toxin beneath the gland (Section 6.2). If the platysma is also treated, injections that go below the mandibular border over the gland need care (Section 4.6).

### 4.5. Depressor Anguli Oris

**What it does.** The depressor anguli oris pulls the mouth corner down. When it is strong or overactive, the corner sits low at rest, the marionette line deepens, and the lower face looks sad or tired. Removing the pull lets the elevators lift the corner.

**Who is a good candidate.** It works best when the muscle tone is high: the corner drops when the patient says “oo”, the marionette line is deepened, or vertical or oblique lines form below the corner of the mouth. But the corner is held by the balance of elevators and depressors, so weakening the depressor raises it in most people, and the indication can be wider than these clear cases.

**Who needs caution.** The corner of the mouth is not held down by the depressor anguli oris alone; jowl sagging and loss of volume above the corner also pull it down. A patient who expects a large lift from depressor BoNT alone needs to be told this before treatment. The change is real but modest, and it is larger when the jowl or the volume is also treated.

**How.** The published doses are 2–4 U per side [31]. The authors use 2–3 U per side, 1 point, at the lower lateral part of the muscle near its origin on the mandible, lateral to the vertical line of the commissure. Of the three muscles in this area—the depressor anguli oris, depressor labii inferioris, and mentalis—the depressor anguli oris is the most superficial. It can be injected superficially, raising a wheal, which keeps the toxin away from the depressor labii inferioris.

**What to watch.** Spread into the depressor labii inferioris gives an asymmetric smile; the superficial, lateral placement above avoids this.

**An observation not previously reported.** When the depressor is switched off, the lip elevators lose their opponent. In a patient whose nasolabial fold is made mainly by strong elevator muscles, the fold can look deeper after depressor anguli oris BoNT. To the authors’ knowledge, this has not yet been described. Such patients should be warned.

**Double use.** Some patients have vertical lines lateral to the marionette line, near the jawline, more oblique toward the outside. The depressor anguli oris and the platysma contribute to these. Treating them is a wrinkle treatment and a contour treatment at the same time.

### 4.6. Platysma

**What it does.** The platysma is a thin sheet that extends from the chest up over the jawline into the lower face, where it runs deep to the depressor anguli oris and inserts into the modiolus and the skin of the lower lip [36,47]. When it is tight, it pulls the lower face down and blurs the jawline. Relaxing it at the level of the jawline removes that pull; the jawline becomes clearer, and the lower face looks lifted. The approach is similar to the Nefertiti lift [11]. The treatment of platysma bands in the neck is a separate indication, with different injection points, and is not covered here.

**Who is a good candidate.** The patient must have a muscle that is pulling. In the only prospective trial of the Nefertiti lift, the treatment was particularly helpful in younger patients with platysma muscle hyperactivity and retained skin elasticity [34]; in that trial, which did not select patients in advance, jawline and jowl changes showed a trend without reaching significance. In the authors’ experience, selecting patients with visible platysma pull and firm skin is what makes the jawline result reliable.

**Who needs caution.** The patient whose jawline is blurred mainly by fat, jowl sagging, or loose skin. Relaxing the platysma will not clear it on its own. The platysma is a free-sliding sheet; it does not produce a facelift-type lift, and expecting one in a loose-skin neck leads to disappointment. These patients need treatment of the fat, the jowl, or the skin, with or without platysma BoNT.

**How.** Published doses vary widely. A published anatomical proposal uses 40 U in total, 2 U per point, along a line 45° outward from the corner of the mouth [8]. The authors inject along the jawline, lateral to the depressor anguli oris point, at or just above the mandibular border, 20–30 U per side, intradermal or subdermal. Deep injection risks the swallowing muscles beneath.

**What to watch.** Some injectors extend platysma injections below the mandibular border into the neck. Two cautions apply there. The first pertains to the total dose: the further down the neck the injections go, the more the total matters. Total doses above 50–60 U are the caution line. Dysphagia and neck-flexor weakness have been reported above 75 U, and severe dysphagia requiring tube feeding for 6 weeks after 60 U [37,38]; a French consensus limits the total to 100 U even for severe bands, with most authors using 30–40 U [37,38]. In band treatment, the approved doses are 26–36 U [32,35], and a meta-analysis of randomized trials in that range found serious adverse events to be rare and similar to placebo [33]. The second caution is related to the submandibular gland. In an older neck with a visible submandibular gland, the gland is covered by the platysma, and relaxing the sheet over it may make a descended gland more visible. Keeping the injections at or above the border avoids this.

Spread into the depressor labii inferioris or risorius gives an asymmetric smile.

**Double use.** As for the depressor anguli oris, vertical and oblique lines above the jawline follow the contraction pattern of the facial part of the platysma and improve with the same injections.

### 4.7. Mentalis

**What it does.** Mentalis BoNT is often called “cobblestone chin BoNT”. For contouring, its more important effect is on chin length. An overactive mentalis pulls the soft tissue of the chin up and in. The chin looks short and pulled back, and the lower face loses its V-line. Relaxing the muscle lets the chin return to its resting length. This has been measured: after mentalis BoNT, the chin point moved downward in 10 of 11 patients [12].

**Who is a good candidate.** A patient whose chin shortens or bunches on lip closure, and whose bone is adequate. The decisive question is bone versus muscle. If the vertical length of the mandible itself is short, filler is the main treatment, and the authors add mentalis BoNT when lip closure shows mentalis overactivity—which is the case in most patients with a short chin—not as a routine for every chin filler. If the patient declines filler—many do—BoNT alone still lengthens the chin, and the authors recommend at least that. If the bone is adequate and the muscle is riding over it, BoNT alone is the best treatment.

**Who needs caution.** A patient whose mentalis is already relaxed—no shortening or bunching on lip closure. There is nothing to release.

**How.** The published doses range from 4 U per side under ultrasound [40] to 12–15 U in total in the measurement study above [12]. The authors use 4–6 U in total, 2 points, low on the chin and near the midline, and find this sufficient in Asian patients. The mentalis has a deep part arising from bone and a superficial part inserting into skin; injection should cover both layers [40,41]. Injections placed too high reach the orbicularis oris; too lateral, the depressor labii inferioris.

**What to watch.** In the first few days after injection, the expression can look slightly unnatural. This is uneven relaxation before the toxin has spread evenly through the muscle, and it settles within days. When it does not settle and a localized bulge appears, the cause is fibers that were missed: the treated fibers relax, and the untreated fibers contract without opposition. This “paradoxical bulging” has been described and resolves with a supplementary injection into the remaining active fibers [39]. Covering the full thickness of the muscle at the first injection prevents it.

The mentalis also supports the lower lip [48]. Rarely, a patient reports after mentalis BoNT that the lips do not close comfortably. In the authors’ experience, this is temporary.

### 4.8. Products and Units

The units of botulinum toxin are product-specific potency units and are not interchangeable between products. The authors use three 100 U botulinum toxin type A products: incobotulinumtoxinA and two Korean products that have no international non-proprietary name (Medytox, Seoul, Republic of Korea). In practice, the authors apply the same unit numbers to these three products; this is common practice in Korea but has not been established by comparative trials, and the doses in this review should not be converted to abobotulinumtoxinA or to any other product. Published doses are quoted with the product named in the source (Table 1).

## 5. Assessment: Choosing the Sites

The seven sites are a checklist, not a menu. Patients rarely know which of them are shaping their face; most know only the square jaw. The physician looks at all seven, explains what is found, and treats the sites that matter. The pairings below, summarized in Table 2, are the ones the authors check in every consultation; they are from the authors’ practice and have not been tested prospectively.

### Sites That Go Together

**The square lower face.** The patient chooses this treatment to get a smaller face or a V-line. The parotid gland and the temporalis serve the same goal and are checked with the masseter (Section 4.1).

**The short chin.** A young patient who needs mentalis BoNT usually has a small mandible. With a small mandible, the jawline is often under-developed as well, and the submandibular gland is then often exposed. The submandibular gland is checked with the chin (Section 4.4).

**The patient who asks for lifting or tightening.** The two depressors, the platysma and the depressor anguli oris, are checked first (Section 4.5 and Section 4.6). The masseter and the parotid are also checked because a wide lower face looks less saggy when it is made lighter. The mentalis is also checked because a short chin makes the lower face look more saggy.

## 6. Safety Points Specific to Contouring

Contouring BoNT differs from wrinkle BoNT in two ways that matter for safety. The doses are larger: a masseter, parotid, submandibular, temporalis, or platysma treatment uses several times the dose of a glabellar treatment, and two or more sites are often treated in one session. Also, two of the seven sites are salivary glands, not muscles. The first difference raises the question of immunogenicity. The second raises questions that most aesthetic physicians have not had to answer before.

### 6.1. Large Doses, Repeated: Immunogenicity

Neutralizing antibodies against BoNT are rare; pooled estimates across therapeutic indications are 1–2%, and in aesthetic use, they are rarer still [49,50]. But some of the known risk factors for antibody formation apply to contouring treatment, in particular a high dose per session and a high cumulative dose; short intervals, booster injections, and formulations with a high protein load are the others [51]. In the Asia-Pacific region, where high-dose contouring is common, secondary non-response has been reported in patients treated with 100 U or more per session [52,53]. Contouring doses are large by nature, so the other factors are the ones to manage. Five rules follow:

1. Treat only the sites that matter, at the lowest dose that produces the desired result. Every unnecessary site adds to the total.
2. Do not give booster injections. If the result is not sufficient, wait for the next cycle.
3. Make the interval as long as the result allows. Once a muscle no longer returns to its original size (Section 4.1), the interval can be very long, and treatment should not be repeated on schedule.
4. Formulations with a lower protein load have been proposed to reduce the risk [50,51]. The evidence is indirect and partly industry-associated, and no product has been shown to prevent antibody formation in contouring use; the choice of product is a matter of judgment, not a recommendation of this review.
5. When a site that used to respond stops responding, think of antibodies before thinking of dose. Confirm with a test injection (frontalis or extensor digitorum brevis test) or a laboratory assay for neutralizing antibodies (mouse hemidiaphragm assay) before increasing the dose [51].

### 6.2. Salivary Glands

Parotid and submandibular BoNT for contouring is widely practiced in East Asia and reported mainly in the Asian literature, but it is off-label everywhere, and the concerns are real: dry mouth, difficulty swallowing, and injecting a structure that most aesthetic physicians have never injected. Recent work from outside Asia has begun to address its safety [54]. This section states the concerns and the answers directly; the site-specific technique is described in Section 4.3 and Section 4.4.

Physiology determines which gland is sensitive. At rest, roughly two-thirds of saliva comes from the submandibular glands; the parotid contributes a minority at rest and most of the flow during eating. Parotid BoNT therefore rarely causes dry mouth. Submandibular BoNT can. The sublingual gland is never a contouring target.

The doses are within an approved range. IncobotulinumtoxinA is approved for chronic sialorrhea at a total of 100 U, given as 30 U to each parotid and 20 U to each submandibular gland [55,56]. The contouring doses in this review—15–40 U per parotid, 10–15 U per submandibular gland—sit inside or close to that range. Up to 250 U in total has been injected into the salivary glands under ultrasound guidance with only mild dry mouth and no dysphagia, facial weakness, or chewing difficulty [57].

The adverse-event data come from patients with too much saliva, and they do not transfer directly to aesthetic patients. Patients with sialorrhea have an excess that the treatment is meant to reduce; aesthetic patients have normal salivary function, and any reduction is a loss. In the pivotal sialorrhea trial and its extension, dry mouth occurred in 4–11% and dysphagia in 1.5–4% of patients [55,56]; a meta-analysis of randomized trials reported 3.3% for each [58]. Whether these rates are higher or lower in aesthetic patients is not known: the subjective consequences of a reduction may differ when baseline function is normal, but no study has measured dry mouth after cosmetic treatment, and the figures should be read with that uncertainty. In the authors’ experience, mild and temporary dry mouth follows submandibular treatment in approximately one in ten patients and almost never follows parotid treatment. A healthy 37-year-old developed severe dysphagia with silent aspiration the day after cosmetic incobotulinumtoxinA, 20 U into each submandibular gland, injected without ultrasound; swallowing recovered over 5 months with rehabilitation [30]. The dose was not high. The problem spread into the swallowing muscles beneath the gland.

Three rules follow.

1. Treat only a gland that shows. Contouring corrects what is seen.

2. Keep the toxin in the gland—with a modest dose, high concentration, and small volume, and no spread downward or deep. This matters most for the submandibular gland, which lies over the swallowing muscles. For the submandibular gland, ultrasound guidance should be the standard precautionary approach for this elective aesthetic treatment: in a cadaver study, blind injection reached the gland in half of attempts and ultrasound-guided injection in over 90% [59], and the reported case of severe dysphagia followed injection without ultrasound [30]. It is especially important for less experienced injectors, in patients with a thick neck or a gland that is hard to feel, and after any previous treatment that caused dry mouth or weakness. This recommendation rests on anatomical accuracy data and expert opinion; there has been no comparative clinical trial. For the parotid, which is large and superficial, palpation-guided injection by an experienced injector is acceptable, and ultrasound is advisable for less experienced injectors.

3. Tell the patient about dry mouth before treatment of the submandibular gland. It is the most common complaint, and it is not a surprise if the patient expected it. After parotid BoNT, dry mouth is rare.

## 7. Limits of This Framework

This is a narrative review. The doses are ranges from the literature and from the authors’ practice, not trial results for every site. The products the authors use, and the limits of unit conversion, are stated in Section 4.8. The evidence is uneven across the seven sites: the masseter has randomized trials, while the temporalis and the submandibular gland have small series and case reports. Two of the seven sites are salivary glands. For them, the aesthetic indication is off-label, and the safety data come mainly from sialorrhea trials in a different patient group.

The authors’ practice is informed by an East Asian population. The muscle and gland volume differ between populations, and so does the idea of a beautiful face: a narrower lower face and a V-line are strong preferences in East Asia but not everywhere. The authors’ doses and their thresholds for treatment should therefore be interpreted as based on one experienced practice in one population, not as a standard for all. The mechanisms, the examination, and the neighbor rule apply everywhere; the numbers may not.

The neighbor effects described in Section 4 are partly demonstrated and partly reasoned. Thickening of the temporalis after masseter treatment has been shown in a randomized trial. The deepening of an elevator-dominant nasolabial fold after depressor anguli oris treatment, and the unmasking of a descended submandibular gland when platysma injections extend below the mandibular border, are clinical reasoning, not measured outcomes.

The framework does not replace bone or volume. When the contour problem is structural—a short mandible, a deficient chin, or true temporal volume loss—filler or surgery is needed, and BoNT works alongside it. The framework identifies these patients so that BoNT is not used where it will not work. It does not treat them.

Finally, the seven-site framework is a proposal based on expert practice. It has not been tested prospectively. Three studies would strengthen the framework: a prospective evaluation of the assessment algorithm against outcomes, identifying clinical predictors of the jawline’s response to platysma BoNT, and measuring changes at neighboring sites after each of the seven treatments.

## 8. Conclusions

Contouring BoNT is a category of its own. Its goal is a change in shape at rest, not the removal of a line. Seven sites serve this goal; the physician looks at all of them and treats the ones that need it. Choosing well depends on three things: examining each site in the position that shows it, confirming that the target is really the cause, and looking at the neighbor site that will change when the target is treated. Used this way, BoNT alone can make the face smaller—by reducing the volume of a muscle or a gland—and make it look lifted—by removing a downward pull—in patients selected for these mechanisms. The seven-site framework is a proposed, expert-based tool for this selection process. Prospective clinical studies are needed to test it, and the doses given here should be interpreted as starting points from one population, not as established values.

## Figures and Tables

**Figure 1:**
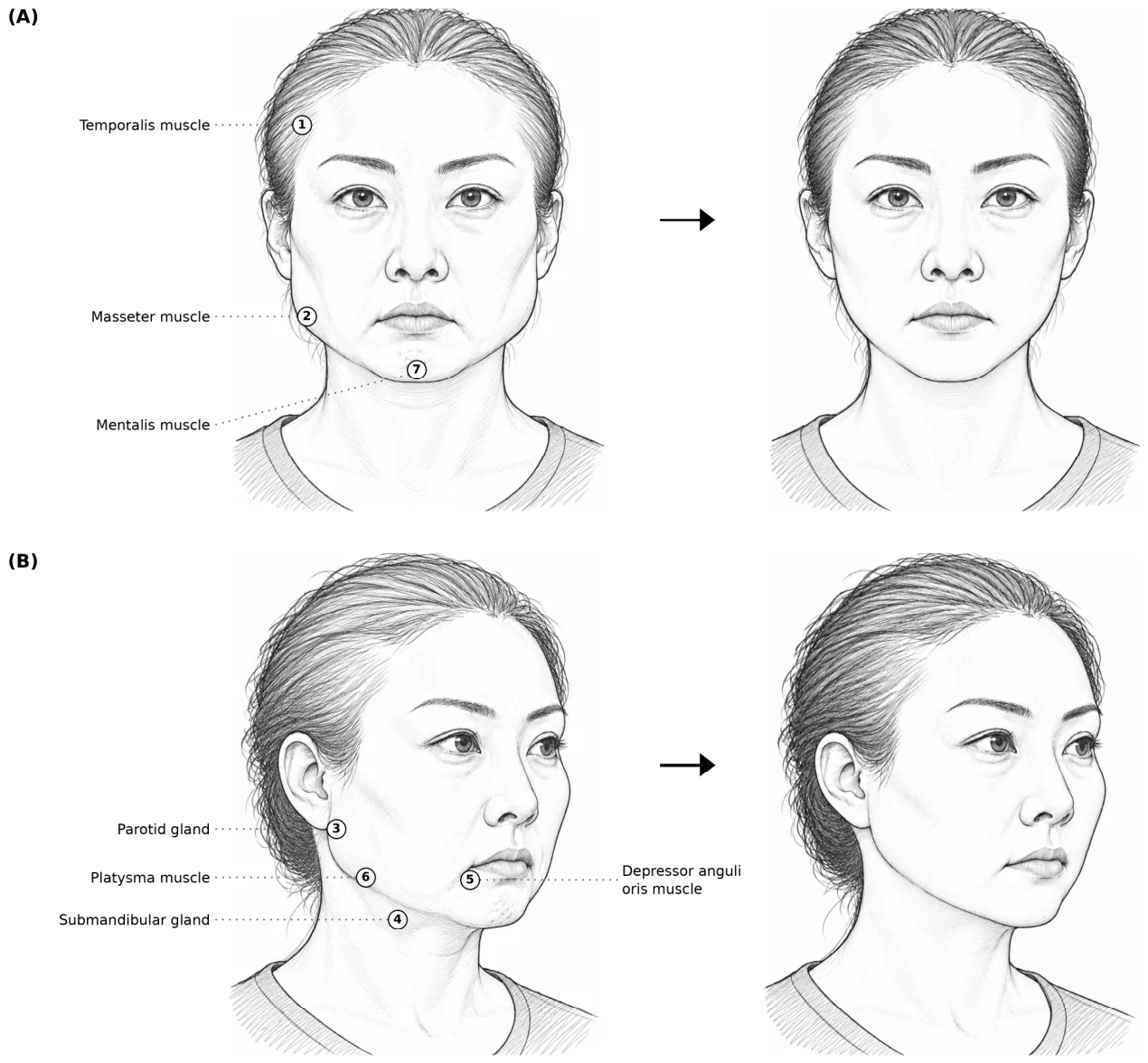
The seven contouring sites and the changes that treatment produces. Schematic illustration, not a patient. (**A**) Frontal view, before (left) and after (right) treatment; sites 1, 2, and 7 are marked. (**B**) Oblique view, before (left) and after (right); sites 3–6 are marked. 1, temporalis muscle; 2, masseter muscle; 3, parotid gland; 4, submandibular gland; 5, depressor anguli oris muscle; 6, platysma muscle; 7, mentalis muscle. The drawings were generated with ChatGPT (GPT-5.6 Sol, OpenAI) from the authors’ specifications and edited by the authors; the anatomical placement of the seven sites was verified by the authors. Only the seven sites differ between the before and after drawings.

**Figure 2:**
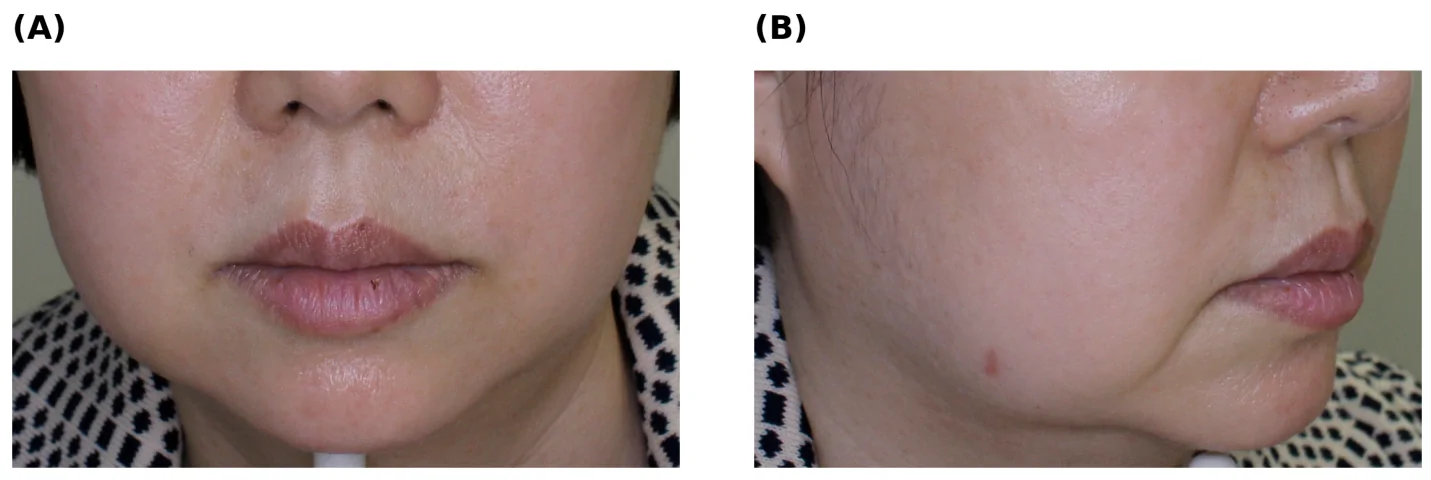
The lateral boundary of the lower face is not always the masseter. Shown is a patient who asked for masseter BoNT because the lower face looked heavy from the front. (**A**) Frontal view. (**B**) 45° view: the lateral boundary is formed by cheek soft tissue in front of the masseter, not by muscle, and is not a BoNT target.

**Figure 3:**
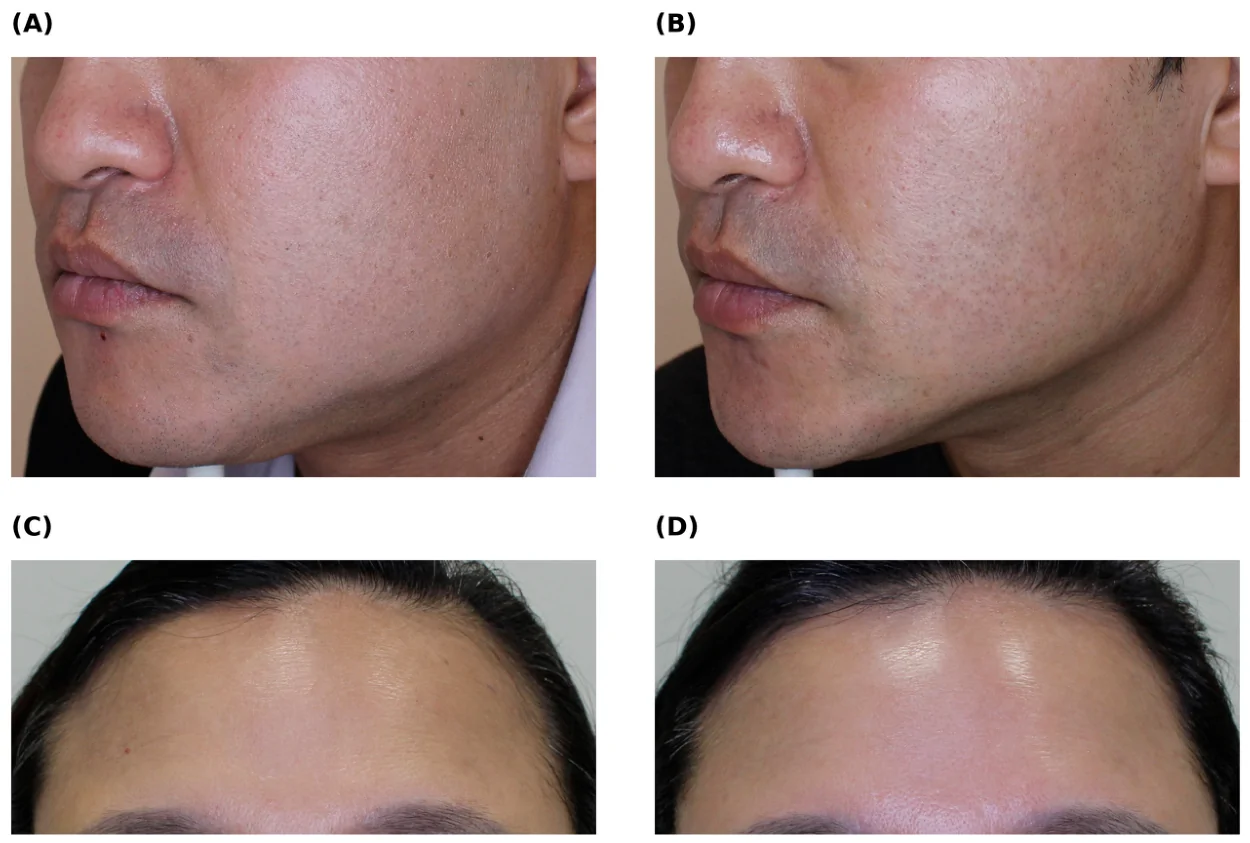
Clinical examples of contouring BoNT. Photographs were taken in the clinic’s photography room with the same camera and standard settings before and after treatment; lighting was not formally standardized, no objective measurements were made, and the examples are illustrative. (**A**,**B**) A man in his forties, 45° view: masseter 10 U per side and parotid 15 U per side; before (**A**) and 7 weeks after (**B**). (**C**,**D**) A woman in her forties, with frontal view of the forehead and temples: temporalis 10 U per side, three treatments about 6 months apart; before the first treatment (**C**) and 3 months after the third (**D**).

**Table 1:** Evidence for each site as a contouring target.

Site	Best Available Evidence for the Contour Effect	Published Doses (Product)	Reported Adverse Events	Evidence Level	Authors’ Starting Dose (Expert Practice, East Asian Patients)
Masseter	Randomized trial [14]; phase 3 program [2]; large series (*n* = 1021) [15]; long-term series [16]; prospective 3D morphometry (*n* = 11, pain population) [17]; reviews [18,19]	Mostly 20–30 U onabotulinumtoxinA per side [18]; abobotulinumtoxinA in [15,16]	Smile asymmetry, chewing fatigue, paradoxical bulging, sunken cheek; temporalis thickening with repeated treatment [14,20]	Randomized trials	10–20 U per side
Temporalis	Prospective series (*n* = 20) [21]; consensus [22]	Varies; see text	Headache, temporal hollowing	Small series/consensus	10–20 U per side
Parotid gland	Randomized trial, high/low dose vs. placebo (*n* = 30) [23]; prospective series (*n* = 33) [24]; series [10]	Intraglandular; doses vary; see text	No serious adverse events in [23,24]; dry mouth uncommon	Randomized trial (level II)	15–40 U per side
Submandibular gland	Prospective series [25]; case series [26]; imaging and anatomy [27,28,29]; case report of severe dysphagia [30]	20 U in 0.2 mL × 5 points, ultrasound-guided [25]; 15 U single point [26]	Dry mouth; dysphagia with spread beneath the gland	Case series/anatomical	10–15 U per side
Depressor anguli oris	Consensus recommendations [31]; anatomical studies	2–4 U onabotulinumtoxinA per side [31]	Asymmetric smile (depressor labii inferioris spread)	Consensus/expert	2–3 U per side
Platysma	Randomized trials for bands [32,33]; prospective study of the jawline (*n* = 30) [34]; case series [11]	Bands: 26–36 U onabotulinumtoxinA [32,35]; jawline: 40 U total, 2 U per point [36]	Dysphagia and neck weakness at high total dose [37,38]; asymmetric smile	Randomized trials (bands); prospective (jawline)	20–30 U per side
Mentalis	Prospective measurement (*n* = 11) [12]; case report [39]; anatomical and ultrasound studies [40,41]	12–15 U onabotulinumtoxinA total [12]; 4 U per side [40]	Lip incompetence, paradoxical bulging	Small series/anatomical	4–6 U total

The doses are for the products named; the units are product-specific and not interchangeable (Section 4.8).

**Table 2:** Sites to check by the patient’s complaint.

The Patient’s Complaint	Sites to Check
A wide face	Masseter muscle, parotid gland, temporalis muscle
A wide upper face (inverted triangle)	Temporalis muscle
A short chin	Mentalis muscle, submandibular gland
A blurred, heavy jawline	Platysma muscle, submandibular gland
Sagging of the lower face	Masseter muscle, parotid gland, mentalis muscle, depressor anguli oris muscle, platysma muscle

## Data Availability

No new data were created or analyzed in this study. Data sharing is not applicable to this article.
